# NopAA and NopD Signaling Association-Related Gene *GmNAC27* Promotes Nodulation in Soybean (*Glycine max*)

**DOI:** 10.3390/ijms242417498

**Published:** 2023-12-15

**Authors:** Yue Wang, Xiaoke Jia, Yansong Li, Shengnan Ma, Chao Ma, Dawei Xin, Jinhui Wang, Qingshan Chen, Chunyan Liu

**Affiliations:** Key Laboratory of Soybean Biology in Chinese Ministry of Education, National Key Laboratory of Smart Farm Technology and System, College of Agriculture, Northeast Agricultural University, Harbin 150030, China; b210301017@neau.edu.cn (Y.W.); s210301806@neau.edu.cn (X.J.); s220301058@neau.edu.cn (Y.L.); a01200201@neau.edu.cn (S.M.); b220301006@neau.edu.cn (C.M.); dwxin@neau.edu.cn (D.X.); wangjinhui113@neau.edu.cn (J.W.)

**Keywords:** soybean, rhizobia, type III effector, NopAA, NopD, signaling association

## Abstract

Rhizobia secrete effectors that are essential for the effective establishment of their symbiotic interactions with leguminous host plants. However, the signaling pathways governing rhizobial type III effectors have yet to be sufficiently characterized. In the present study, the type III effectors, NopAA and NopD, which perhaps have signaling pathway crosstalk in the regulation of plant defense responses, have been studied together for the first time during nodulation. Initial qRT-PCR experiments were used to explore the impact of NopAA and NopD on marker genes associated with symbiosis and defense responses. The effects of these effectors on nodulation were then assessed by generating bacteria in which both *NopAA* and *NopD* were mutated. RNA-sequencing analyses of soybean roots were further utilized to assess signaling crosstalk between NopAA and NopD. *NopAA* mutant and *NopD* mutant were both found to repress *GmPR1*, *GmPR2*, and *GmPR5* expression in these roots. The two mutants also significantly reduced nodules dry weight and the number of nodules and infection threads, although these changes were not significantly different from those observed following inoculation with double-mutant (HH103Ω*NopAA&NopD*). *NopAA* and *NopD* co-mutant inoculation was primarily found to impact the plant–pathogen interaction pathway. Common differentially expressed genes (DEGs) associated with both NopAA and NopD were enriched in the plant–pathogen interaction, plant hormone signal transduction, and MAPK signaling pathways, and no further changes in these common DEGs were noted in response to inoculation with HH103Ω*NopAA&NopD*. *Glyma.13G279900* (*GmNAC27*) was ultimately identified as being significantly upregulated in the context of HH103Ω*NopAA&NopD* inoculation, serving as a positive regulator of nodulation. These results provide new insight into the synergistic impact that specific effectors can have on the establishment of symbiosis and the responses of host plant proteins.

## 1. Introduction

Through their symbiotic interactions with leguminous plants, rhizobia can facilitate biological nitrogen fixation that is conducive to more robust plant growth and development, reducing the need to apply chemical fertilizers to crops [1,2]. Soybeans are the most widely cultivated legume in the world, serving as a key source of oil and protein for human consumption [3]. Large quantities of industrial nitrogen fertilizer are routinely applied to meet the levels of soybean production necessary to meet with current demand [4]. However, this fertilizer application poses a serious threat to environmental integrity while also threatening the diversity of soil microbes in affected regions [5,6,7]. Research focused on interactions between soybean plants and rhizobia can profile a foundation for the rational application of biological nitrogen fixation, thus providing a more efficient and ecologically sound supply of nitrogen to growing plants [8,9].

The establishment of the symbiotic association between nitrogen-fixing rhizobia and their leguminous hosts is a complex process that necessitates frequent reciprocal signaling between these microbes and the host plants [10]. Soybean-derived flavonoids can promote rhizobial synthesis and secretion of nodulation factor (NF) via the upregulation of the *NodD* gene [11,12]. Once activated, NodD binds to the promoter upstream of *TtsI*, inducing the expression of this gene [13]. TtsI is capable of binding to *Tts box* promoter sequences, thereby inducing the upregulation of type III secretion system (T3SS)-related genes [14]. The type III effectors (T3Es) that are secreted by these T3SS systems are closely associated with successful rhizobial colonization. According to the results of the current study, T3Es can be divided into two types: one is structural proteins and the other is nodulation outer proteins (Nops). Indeed, *TtsI* mutants exhibit markedly suppressed T3E synthesis and release, thus adversely impacting the establishment of symbiotic relationships with host plants. Transcriptomic analyses, extracellular protein characterization, and nodulation testing have all confirmed that the *TtsI* mutant strain of *Sinorhizobium fredii* HH103 exhibits impaired T3Es expression and nodulation attributable to this *TtsI* mutation. In addition to their effects on the establishment of symbiotic relationships, T3Es also govern the induction of plant defense responses, thereby influencing rhizobia colonization efficiency [15,16,17]. *GmPR1* (pathogenesis-related gene 1) expression was significantly elevated in the roots of Williams 82 soybeans following inoculation with the *TtsI* mutant strain as compared to the wild type strain [18]. Research focused on pathogenic bacteria such as *Pseudomonas* and *Xanthomonas* has additionally demonstrated an essential role for T3Es in the incidence of infection [19,20].

To date, a subset of Nop proteins have been identified and subject to biochemical and functional characterization. For example, the T3SS apparatus of *Sinorhizobium* has been shown to consist of at least three members of this protein family: NopA, NopB, and NopX [21]. The *Bradyrhizobium* USDA110 NopP protein is capable of interacting with the R protein GmNNL1 and promoting the induction of immune responses in hosts [22]. The E3 ubiquitin NopM may function by regulating the activity of the MAPK pathway during the establishment of symbiotic relationships [23]. The glycoside hydrolase 12 (GH12) family glycosyl hydrolase NopAA can hydrolyze the cell wall components β-glucan and xyloglucan into sugars, thus favoring rhizobia infection [24]. It can further influence nodule numbers by shaping the number of infection threads, differentially impacting the nodulation of soybean cultivars with distinct genetic characteristics [24].

The functional importance of T3SS activity in the context of symbiosis is generally not dependent on any specific T3E, instead arising from the redundant, synergistic, or antagonistic interactions of various T3Es [25,26]. Deleting a given T3E may thus enhance or inhibit nodulation [9,27], but it may also have no impact on this process. Deleting *Bel2-5*, *ErnA*, *NopAB*, *NopC*, *NopD*, *NopE*, *NopF*, *NopI*, *NopJ*, *NopL*, *NopM*, *NopP*, or *InnB* can reportedly inhibit nodulation [28,29,30,31], but the impact of specific T3Es on the process of nodule formation is also believed to be host plant-dependent [32,33,34,35]. For example, deleting *NopT* in *Ensifer fredii* strain NGR234 can interfere with *Tephrosia vogelii* nodule formation while enhancing such nodulation in *Crotalaria juncea* [36]. Particular T3Es can interact in concert to shape symbiotic nodulation. For example, the double mutation of *NopL* and *NopP* can interfere with nodulation in *Flemingia congesta* to a greater degree than the single mutation of either of these Nop genes in NGR234 [37]. Similarly, the double mutation of *nopP1* and *nopM1* in *Bradyrhizobium vignae* ORS3257 had a more pronounced impact on nodule formation [30,38]. While NopT and NopP reportedly serve as respective promoters and inhibitors of nodulation, the inoculation of soybean plants with the HH103Ω*NopT&NopP* strain has been shown to result in the formation of fewer nodules than inoculation with the HH103Ω*NopT* strain [39]. RNA-seq analyses have also highlighted a potential role for GmPBS1, which interacts with HH103-derived NopT, in the signaling crosstalk between NopT and NopP [39]. No differences in nodule formation were observed when comparing HH103Ω*NopL&NopT* inoculation to HH103Ω*NopL* or HH103Ω*nopP* [40]. QTL and RNA-seq analyses have also highlighted several NopT and NopL-related genes [40].

Prior RNA-seq and QTL screening experiments have identified a number of NopAA-regulated genes in soybean plants, including the defense response-related *GmPR1* gene as well as members of the ethylene response factor (*ERF*) and *WRKY* transcription factor families [41,42,43]. NopD is a positive regulator of nodulation first identified in *S. fredii* HH103 culture supernatants [44]. It harbors a C-terminal structural domain homologous to the ubiquitin-like proteinase Ulp1, and the homologous *Xanthomonas* protein XopD can interact with small ubiquitin-like modifier (SUMO)-binding proteins to facilitate the removal of SUMO-binding plant proteins, indicating that NopD may play a similar role [45]. In *Arabidopsis thaliana*, XopD can suppress immune response activity [46], whereas NopD reportedly promotes programmed cell death in tobacco [44]. The NopD homolog *Bradyrhizobium elkanii* T3E Bel2-5 can further regulate nodule numbers and the differential expression of a wide range of redox-related genes [28]. The ability of NopD, XopD, and Bel2-5 to interact with host plants is closely tied to the ULP1 domain [28,45,47]. Much as with NopAA, mutations in Bel2-5 modulate the expression of several soybean genes including *ERF1b*, *ERF98*, and *WRKY33/75* [28]. These results suggest that both NopAA and NopD may be involved in the regulation of plant defense responses during the establishment of symbiosis and may be related in some signaling pathways.

In the present study, the inoculation of soybean plants with both *NopAA* mutant and *NopD* mutant strains was found to reduce the expression of the defense-related *GmPR1*, *GmPR2*, and *GmPR5* following rhizobial infection. While HH103Ω*NopAA&NopD* inoculation similarly reduced soybean nodulation, it did so to an extent that was not significantly different from that observed for HH103Ω*NopAA* or HH103Ω*NopD*. RNA-seq analysis revealed that the expression of a range of plant–pathogen interaction, plant hormone signal transduction, and MAPK signaling pathway-related genes was similarly impacted by NopAA and NopD. The NAC family transcription factor *Glyma.13G279900* (*GmNAC27*) was subsequently identified through WGCNA and qRT-PCR experiments as a candidate gene associated with these two T3Es. Transgene analyses then revealed that *GmNAC27* was responsive to the co-associative effects of NopAA and NopD, serving as a positive regulator of nodulation activity. Together, these results provide new insight into the mechanistic basis for legume–rhizobia interactions.

## 2. Results

### 2.1. HH103ΩNopAA&D Inhibits Nodule Formation

**Figure 1:**
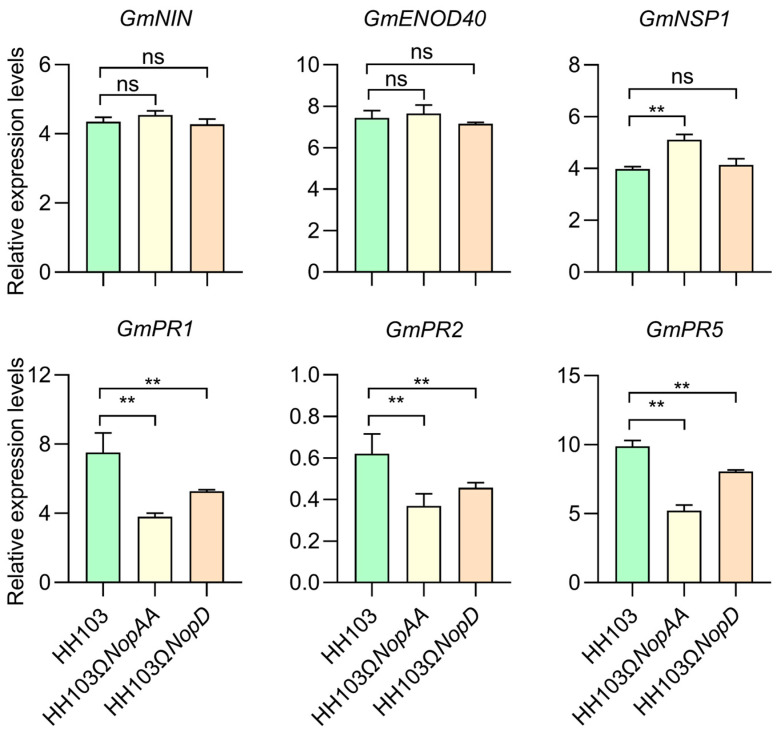
The impact of HH103 and mutants’ inoculation of nodulation-related gene expression. The relative expression levels of symbiosis-related genes (*GmNIN*, *GmENOD40*, and *GmNSP1*) and defense-related genes (*GmPR1*, *GmPR2*, and *GmPR5*) were identified, and the 2^−ΔΔCt^ method was used to calculate relative gene expression, with *GmUNK1* (*Glyma.12g020500*) serving as an internal control gene. The calibration samples were SN14 roots inoculated with MgSO_4_ and used for normalization. Results are means ± SEM from three replicates. Significance was determined by multifactorial analysis of variances (ANOVAs), “**” represent significant differences (*p* < 0.05), while “ns” indicate no significant differences.

The ability to successfully evade the host defense response is important for successful colonization of rhizobia [48]. Similar to pathogenic bacteria, rhizobia utilize type III effectors to suppress host defenses or activate symbiotic responses. To analyze the host signaling pathways in which the type III effectors NopAA and NopD are mainly involved, in the present study the changes in symbiosis-related genes (*GmNIN*, *GmENOD40*, and *GmNSP1*) and defense-related genes (*GmPR1*, *GmPR2*, and *GmPR5*) expression in SN14 following HH103, HH103Ω*NopAA*, or HH103Ω*NopD* inoculation were next assessed at 36 h post-infection [1,49]. In these analyses, significantly lower levels of *GmPR1*, *GmPR2*, and *GmPR5* expression were detected in SN14 plants following *NopAA* mutant or *NopD* mutant inoculation as compared to parental HH103 inoculation (Figure 1). The *NopAA* mutant also induced higher levels of *GmNSP1* expression (Figure 1). This suggests that both of these T3Es may play a role in the establishment of symbiosis through their ability to regulate defensive responses.

**Figure 2:**
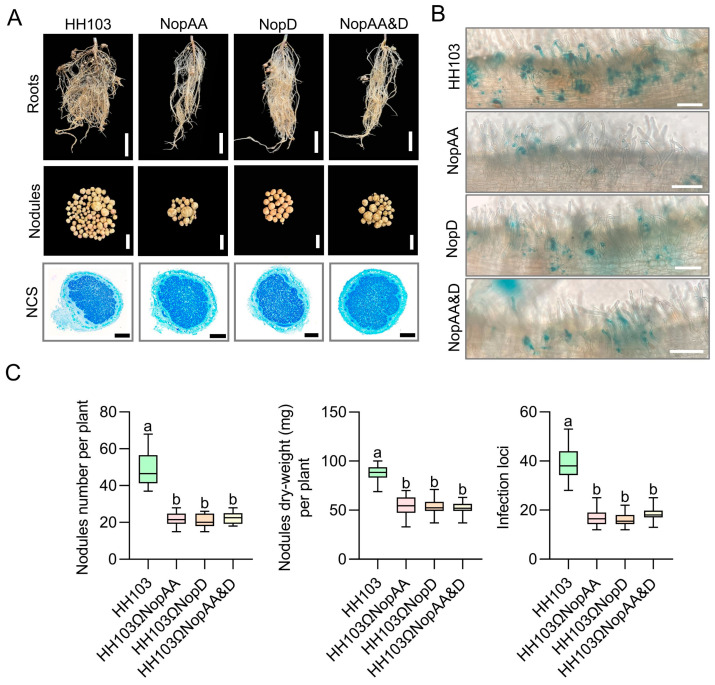
Comparisons of SN14 nodule phenotypes induced by inoculation with the *NopAA*, *NopD*, and *NopAA&D* mutant and HH103 strains. (**A**) SN14 nodule phenotypes following HH103, HH103Ω*NopAA*, HH103Ω*NopD*, and HH103Ω*NopAA&D* inoculation. Scale bars: 5 cm (roots), 2 mm (nodules), 50 μm (nodule cross-sections [NCS]). (**B**) Infection thread numbers in SN14 samples following HH103, HH103Ω*NopAA*, HH103Ω*NopD*, and HH103Ω*NopAA&D* inoculation. Scale bars: 200 μm. (**C**) Quantitative analyses corresponding to nodules number, dry weight, and infection threads number in SN14 samples following inoculation with HH103, HH103Ω*NopAA*, HH103Ω*NopD*, and HH103Ω*NopAA&D*. Data are presented as the averages of three biological replicates (*n* = 20 plants/replicate). Significance was determined by multifactorial analysis of variances (ANOVAs), different letters represent significant differences (*p* < 0.05), while the same letters indicate no significant differences.

To build on these results, *NopD* was next mutated in the HH103Ω*NopAA* strain to yield the HH103Ω*NopAA&D* mutant strain, with SN14 nodule phenotypes then being evaluated following inoculation with this strain, single mutant strains, or parental HH103. Significant reductions in number of nodules and nodules dry weight were observed following HH103Ω*NopAA*, HH103Ω*NopD*, or HH103Ω*NopAA&D* inoculation as compared to HH103 inoculation (Figure 2A,C). However, no differences in these values were noted when comparing the single- and double-mutant HH103 strains (Figure 2A,C). Toluidine blue staining of nodule cross-sections (NCS) similarly revealed no significant differences in infected cell density within these nodules (Figure 2A). Infection thread phenotypes were additionally evaluated following inoculation with these different HH103 strains expressing the *GUS* reporter gene. Significant reductions in infection thread numbers were noted following SN14 inoculation with the *NopAA* mutant, *NopD* mutant, or *NopAA&D* mutant strains as compared to HH103 inoculation (Figure 2B,C). This suggests that both NopAA and NopD can suppress nodulation by inhibiting rhizobial infection. Consistently, no significant differences in the impact of *NopAA* mutant, *NopD* mutant, or *NopAA&D* mutant inoculation were observed on infection threads events (Figure 2B,C). This suggests that the *NopAA* and *NopD* co-mutant inoculation does not further inhibit nodulation compared to *NopAA* mutant or *NopD* mutant, which means that NopAA and NopD might exist as redundant functions in nodulation.

### 2.2. Similar Patterns of Differential Gene Expression Are Evident in Soybean Roots following HH103ΩNopAA and HH103ΩNopD Inoculation

**Figure 3:**
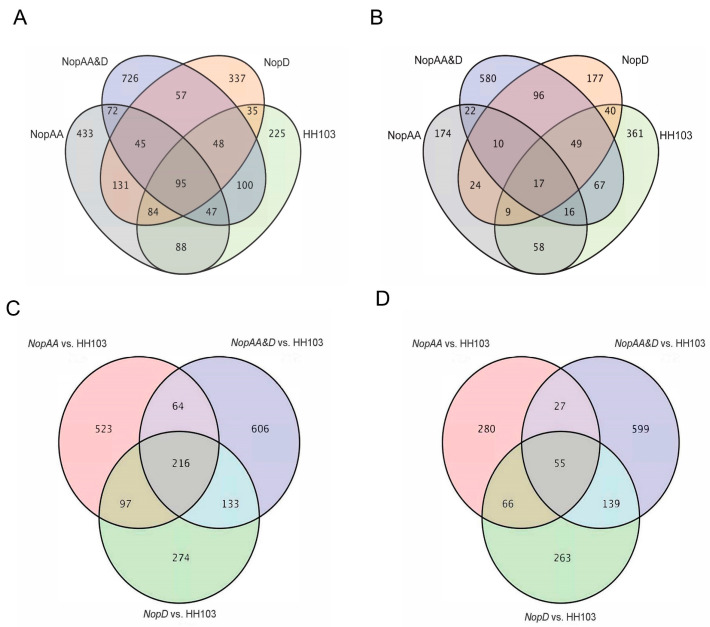
Venn diagrams representing the numbers of DEGs identified in SN14 roots. (**A**) Downregulated genes with the indicated rhizobial strains as compared to mock inoculation. (**B**) Upregulated genes with the indicated rhizobial strains as compared to mock inoculation. (**C**) Downregulated genes with different HH103 mutant strains relative to parental HH103 inoculation. (**D**) Upregulated genes with different HH103 mutant strains relative to parental HH103 inoculation.

To further explore the signaling crosstalk between NopAA and NopD, RNA-seq analyses of roots collected from SN14 plants inoculated with HH103, HH103Ω*NopAA*, HH103Ω*NopD*, HH103Ω*NopAA&D*, or MgSO_4_ (mock control) were next conducted. Relative to mock treatment, the roots of plants inoculated with HH103, HH103Ω*NopAA*, HH103Ω*NopD*, and HH103Ω*NopAA&D* exhibited 772, 995, 832, and 1190 downregulated differentially expressed genes (DEGs), respectively (Figure 3A). In addition, 355 common downregulated DEGs were identified when comparing *NopAA*-mutant-inoculated and *NopD*-mutant-inoculated roots (Figure 3A), while 259 and 245 overlapping downregulated DEGs were identified when comparing inoculation with HH103Ω*NopAA* or HH103Ω*NopD* and HH103Ω*NopAA&D*, respectively (Figure 3A). Relative to mock treatment, the roots of plants inoculated with HH103, HH103Ω*NopAA*, HH103Ω*NopD*, and HH103Ω*NopAA&D* exhibited 617, 330, 422, and 857 upregulated DEGs (Figure 3B), including 60, 65, and 172 common upregulated DEGs when comparing roots inoculated with *NopAA* mutant and *NopD* mutant, *NopAA* mutant and *NopAA&D* mutant, and *NopD* mutant and *NopAA&D* mutant strains, respectively (Figure 3B).

To examine the impact of NopAA and NopD on gene expression in soybean roots, DEGs induced by inoculation with the HH103Ω*NopAA*, HH103Ω*NopD*, and HH103Ω*NopAA&D* were identified through a comparison with HH103-inoculated roots. This approach revealed that inoculation with the *NopAA* mutant, *NopD* mutant, and *NopAA&D* mutant strains was associated with 1328 (428 upregulated, 900 downregulated), 1243 (523 upregulated, 720 downregulated), and 1839 (820 upregulated, 1019 downregulated) DEGs, respectively (Figure 3C,D and Figure S1). Based on these results, it appears that HH103Ω*NopAA* and HH103Ω*NopD* inoculation induces highly overlapping DEG profiles in soybean roots, while HH103Ω*NopAA&NopD* inoculation induces even higher numbers of DEGs when comparing gene expression profiles to those induced by Mock or HH103 inoculation.

### 2.3. NopAA and NopD Co-Mutation Primarily Impacts the Plant–Pathogen Interaction Pathway

**Figure 4:**
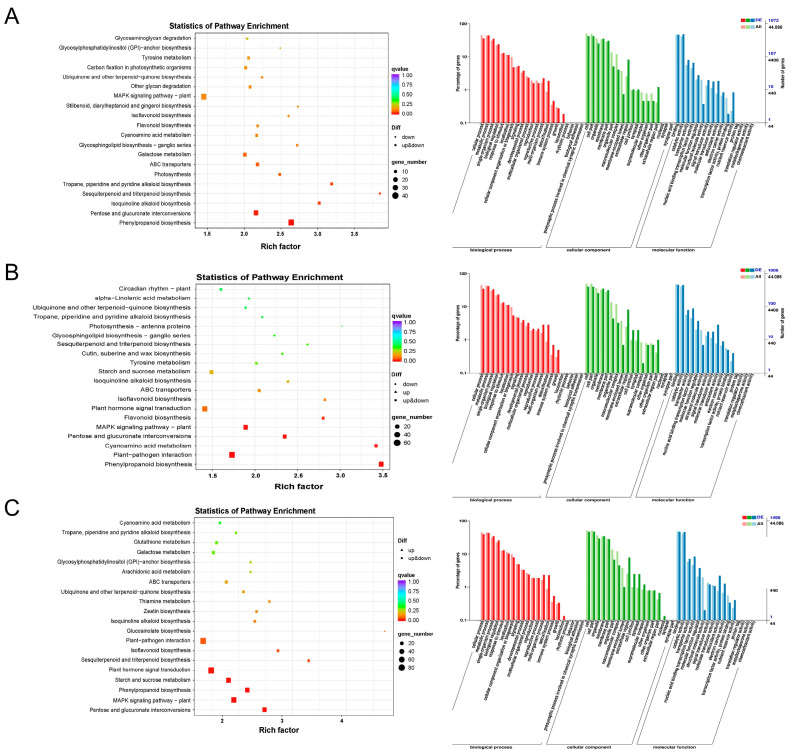
Functional analyses of genes differentially regulated by different mutant HH103 strains. (**A**–**C**) KEGG and GO enrichment analysis results for genes differentially expressed when comparing HH103Ω*NopAA* (**A**), HH103Ω*NopD* (**B**), and HH103Ω*NopAA&D* (**C**) inoculation to HH103 inoculation.

KEGG and GO enrichment analyses were performed on DEGs to determine their signaling pathways and basic functions. Considerable number of DEGs related to NopAA were mainly enriched in phenylpropanoid biosynthesis, MAPK signaling and pentose and glucuronate interconversion signaling pathways (Figure 4A). NopD mainly affects genes enriched in the plant–pathogen interaction, plant hormone signaling transduction, and phenylpropanoid biosynthesis pathways (Figure 4B). Compared with HH103, the inoculation of the HH103Ω*NopAA&NopD* caused differences in the expression of genes enriched mainly in plant–pathogen interaction, MAPK signaling pathway, and plant hormone signal transduction pathways (Figure 4C). And these DEGs clusters were assigned to three broad categories: biological processes, cellular components, and molecular functions; most genes in these categories were enriched in cellular processes, metabolic process, and single-organism processes; cells and cell parts and organelles; and binding, catalytic activity, and transporter activity, respectively (Figure 4A–C).

### 2.4. NopAA and NopD Have Similar Effects on the Plant–Pathogen Interaction Pathway

**Figure 5:**
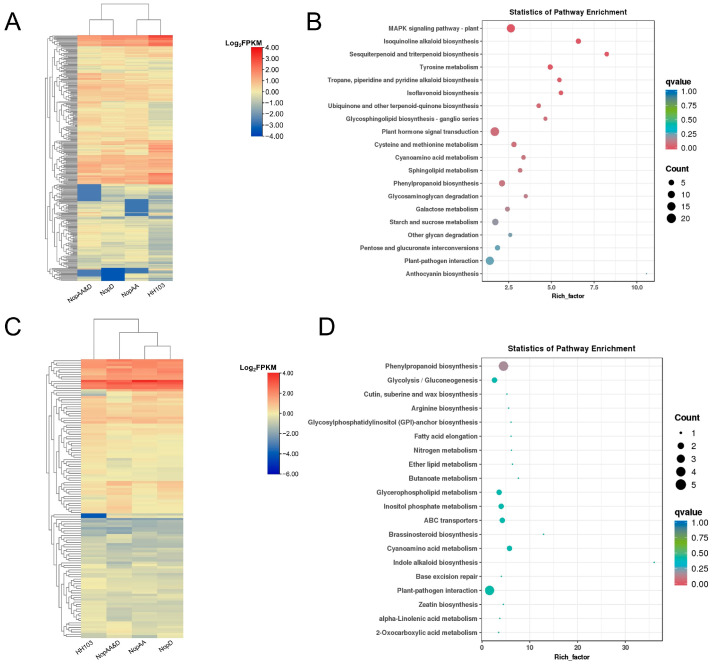
Analyses of common DEGs overlapping between the NopAA vs. HH103 and NopD vs. HH103 comparisons. (**A**,**B**) Heatmap (**A**) and KEGG enrichment analyses (**B**) of common downregulated DEGs. (**C**,**D**) Heatmap (**C**) and KEGG enrichment analyses (**D**) of common upregulated DEGs.

To better understand the signaling crosstalk between NopAA and NopD, the overlapping DEGs between the HH103Ω*NopAA* vs. HH103 and HH103Ω*NopD* vs. HH103 comparisons were next identified. Relative to HH103Ω*NopAA* and HH103Ω*NopD* inoculation, HH103Ω*NopAA&NopD* inoculation did further impact the expression of the 434 common DEGs (121 upregulated, 313 downregulated) (Figure 3A,B and Figure 5A,C). KEGG enrichment analyses of the common upregulated DEGs revealed that they were primarily enriched in the phenylpropanoid biosynthesis and plant–pathogen interaction signaling pathways (Figure 5D), whereas many of the common downregulated DEGs were enriched in the MAPK signaling, plant hormone signal transduction, and plant–pathogen interaction pathways (Figure 5B). Strikingly, several of these common DEGs are also associated with XopD, which is also released by bacterial secretion systems (Figure S2). These data highlight potential crosstalk between NopAA and NopD with respect to their effects on downstream plant–pathogen interaction, MAPK signaling, and plant hormone signal transduction activity in soybean roots.

### 2.5. WGCNA Analyses Reveal Candidate NopAA and NopD-Associated Genes

**Figure 6:**
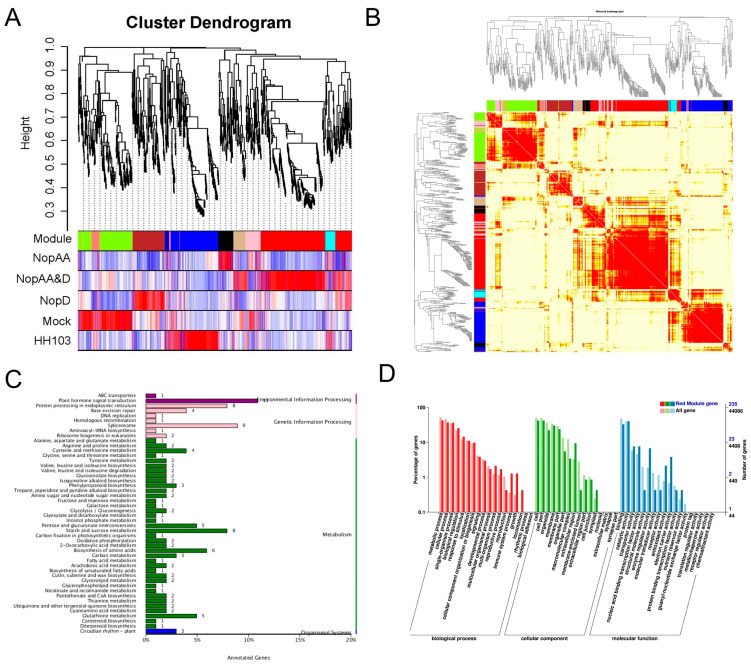
Transcriptomic WCGNA Analyses. (**A**) Component analysis of the module corresponding to included genes. (**B**) Gene co-expression network heatmap. (**C**,**D**) KEGG (**C**) and GO (**D**) enrichment analyses of genes included in red modules.

While *NopAA* and *NopD* co-mutant inoculation did not significantly impact the overlapping DEGs associated with these effectors, a number of DEGs were also identified in soybean roots in response to HH103Ω*NopAA&D* inoculation. To better clarify the genes that were responsive to *NopAA* and *NopD* co-mutant inoculation, all RNA-seq data were next utilized to conduct a weighted correlation network analysis (WGCNA). In total, 1428 genes were grouped into nine co-expressed gene clusters based upon their correlational relationships, and these modules were assigned different colors (Figure 6A,B). Correlations among modules were evaluated with a module eigengene adjacency heatmap, which revealed that genes in the red module were specifically expressed in the roots following HH103Ω*NopAA&D* inoculation (Figure 6A). KEGG enrichment analyses indicated that the genes in the red module were primarily enriched in the plant hormone signal transduction pathway (Figure 6C). In terms of GO term enrichment, these red module genes were primarily enriched in the “metabolic process, cellular processes, and single-organism processes” biological process terms, the “cells and cell parts and organelles” cellular component terms, and the “binding, catalytic activity, and transporter activity” molecular function terms (Figure 6D). Analyses of the FPKM values for the hub genes in this red module were conducted (Figure S3), revealing that these genes were significantly repressed in roots inoculated with HH103 & *NopAA* (Table S3).

### 2.6. qRT-PCR Verification of Candidate Genes

**Figure 7:**
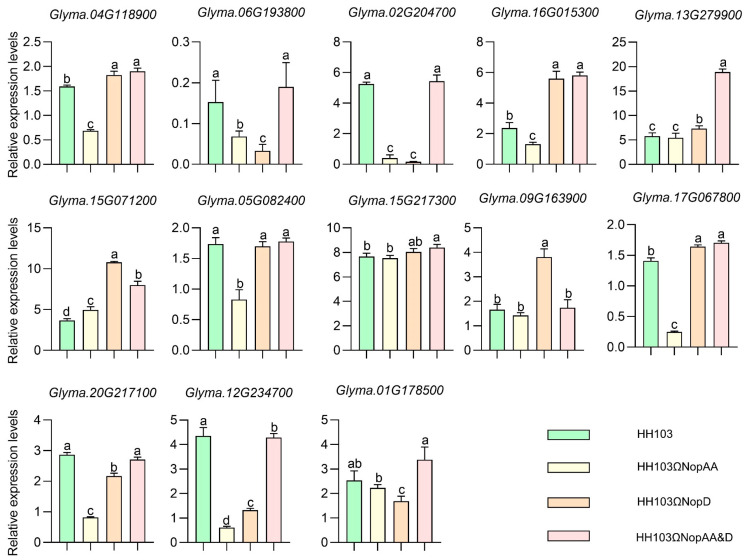
qRT-PCR-based validation of NopAA&D mutant-induced hub genes expression in soybean roots. *GmUNK1 (Glyma.12g020500)* served as an internal control gene. The calibration samples were SN14 roots inoculated with MgSO_4_ and used for normalization. Data are presented as means with standard deviations. Significance was determined by multifactorial analysis of variances (ANOVAs), different letters represent significant differences (*p* < 0.05), while the same letters indicate no significant differences.

Next, the hub genes identified within the red module were annotated, and relative changes in their expression were assessed in the roots of soybean plants following inoculation with mutant or parental HH103 strains. Of the analyzed genes, *Glyma.13G279900* was found to be upregulated to a significantly higher level in roots inoculated with HH103Ω*NopAA&D* as compared to roots inoculated with HH103 (Figure 7), whereas its expression was not significantly impacted by inoculation with HH103Ω*NopAA* as compared to HH103, although it was significantly upregulated in HH103Ω*NopD*-inoculated roots. No other analyzed genes exhibited significant upregulation in response to HH103Ω*NopAA&D* inoculation.

### 2.7. Glyma.13G279900 Is a NAC Family Transcription Factor Located on the Cell Nucleus

*Glyma.13G279900* encodes the NAC family transcription factor GmNAC27. Phylogenetic analyses revealed a close evolutionary relationship between GmNAC27 and similar genes in *A. thaliana* (Figure S4). A subcellular localization analysis of GmNAC27 conducted by infiltrating *Nicotiana benthamiana* leaves wit*h Agrobacterium tumefaciens EHA105* harboring the *CaMV35S: GmNAC27:GFP* fusion vector revealed that the GmNAC27-GFP fusion protein is primarily localized to the nucleus in infected cells (Figure S5).

### 2.8. Analyses of the Impact of GmNAC27 RNA-Interference and Overexpression on Soybean Nodulation

**Figure 8:**
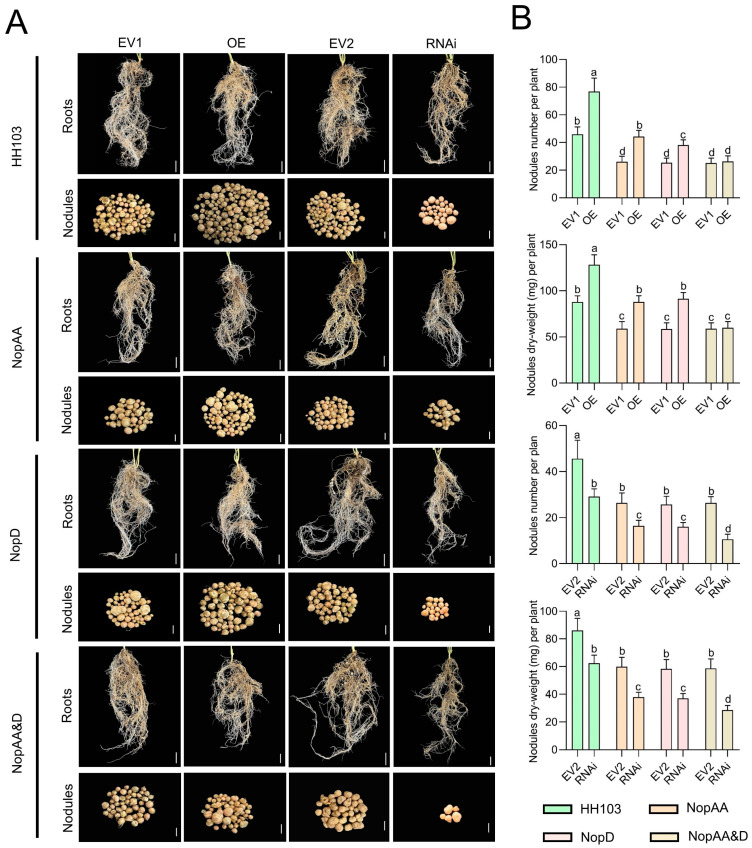
Nodule phenotypes associated with the overexpression or RNAi of *GmNAC27*. (**A**) Nodule phenotypes for hairy roots transformed with EV1, OE, EV2, and RNAi constructs following HH103, NopAA mutant, NopD mutant, or NopAA&D mutant inoculation. EV1, Empty vector for *GmNAC27* overexpression; OE, *GmNAC27* overexpression vector under the control of CaMV35S; EV2, Empty vector for RNAi; RNAi, *GmNAC27* silencing. Scale bars: 1 cm (roots), 2 mm (nodules). (**B**) Quantification of nodules numbers and dry weight. Data are presented as the averages of three biological replicates (*n* = 20 plants/replicate). Significance was determined by multifactorial analysis of variances (ANOVAs), different letters represent significant differences (*p* < 0.05), while the same letters indicate no significant differences.

To establish the impact of GmNAC27 on symbiotic nodulation in soybean plants, this gene was next silenced or overexpressed via soybean transgenic hairy root transformation using *Agrobacterium rhizogenes* strain *K*599 carrying the *pB7GWWIWG2(II)-DsRed-GmNAC27* or *pSoy10-GmNAC27-GFP* plasmid constructs, respectively. Successful *GmNAC27* overexpression or RNAi was confirmed via qRT-PCR (Figure S6). Hairy roots overexpressing *GmNAC27* exhibited significantly higher numbers of nodules and nodule dry weight relative to control plants following HH103, *NopAA*, or *NopD* single mutant inoculation, with nodulation remaining more robust following parental HH103 inoculation as compared to inoculation with either mutant strain (Figure 8). However, no significant differences in nodule number or dry weight were noted when comparing *GmNAC27*-OE hairy roots inoculated with HH103Ω*NopAA&D* and the corresponding EV1 empty vector control (Figure 8). Significant reductions in nodule number and dry weight were observed for hairy roots in which *GmNAC27* had been silenced following HH103, HH103Ω*NopAA*, HH103Ω*NopD*, or HH103Ω*NopAA&D* inoculation as compared to corresponding EV2 control plants (Figure 8). Analyses of symbiosis and defense marker gene expression in transgenic roots were also assessed, revealing that *GmNAC27* had no effect on symbiosis marker genes or *GmPR2*, whereas it was able to positively regulate *GmPR1* and *GmPR5* expression (Figure S7). These results suggest that GmNAC27 may play a role in the signaling crosstalk between the T3Es NopAA and NopD through its ability to regulate defense responses and to serve as a positive regulator of nodulation.

## 3. Discussion

The initial analyses conducted herein confirmed that NopAA and NopD similarly impacted the expression of the *GmPR1*, *GmPR2*, and *GmPR5* marker genes during rhizobia infection, while *NopAA* and *NopD* co-mutant inoculation had no significant effects on nodule formation beyond those observed in the context of inoculation with mutant strains for either of these genes individually. Relative to HH103 inoculation, the inoculation of soybean plants with the *NopAA* mutant and *NopD* mutant strains resulted in the differential expression of a large number of genes associated with the plant–pathogen interaction, plant hormone signal transduction, and MAPK signaling pathways. GmNAC27 was ultimately identified as a soybean protein that was differentially expressed in response to the concurrent deletion of *NopAA* and *NopD*. Together, these results offer a new foundation for research focused on clarifying the mechanistic crosstalk between signaling pathways and associated regulatory mechanisms associated with the function of NopAA and NopD in the establishment of host–rhizobia symbiosis.

First characterized through studies of pathogenic bacteria, T3SS-mediated T3E secretion can enable these pathogens to evade plant immune responses [50]. Plants have evolved the ETI response to T3E exposure, which typically manifests in the form of a robust hypersensitivity reaction resulting in cell death [51]. Rhizobia-derived T3Es often regulate immune functionality in host plants in addition to supporting the establishment of symbiosis [52]. In this study, the relative levels of nodulation maker genes (*GmNIN* [53], *GmENOD40* [54], *GmNSP1* [55]) and defense-related genes (*GmPR1* [8], *GmPR2*, *GmPR5*) were assessed to better clarify the effects of NopAA and NopD on host genes expression. Inoculation with both the *NopAA* mutant and the *NopD* mutant strains resulted in significant reductions in *GmPR1*, *GmPR2*, and *GmPR5* expression, with this suppression being most pronounced for the *NopAA* mutant. This stronger impact of NopAA on host defense gene expression may be related to its ability to hydrolyze cell walls, thereby inducing more robust immune response activity [24]. The fact that NopD regulates defensive responses via programmed cell death pathways may also contribute to this observation [44]. Together, these findings suggest that NopAA and NopD primarily play roles in the modulation of defensive responses in soybean roots, rather than directly influencing nodulation-related symbiotic signals.

The effects of T3SS activity in the context of symbiotic interactions are not dependent on the effects of a single T3E, instead arising as a result of redundant, synergistic, or antagonistic effects among multiple T3Es [26]. With the exception of the complete inhibition of Rj2-soybean nodulation in response to NopP in USDA122, there have not been any reports of any one T3E completely determining whether or not nodulation can occur [56]. In previous studies, our team has utilized HH103 T3E gene insertion mutants and a variety of soybean genetic resources to assess nodulation dynamics, revealing soybean variety-specific effects of these T3Es [21,24,44]. Both NopAA and NopD were previously found to be likely contributors to the nodulation process. To better understand how NopAA and NopD impact nodulation, the HH103Ω*NopAA&NopD* strain was generated in this present study. HH103Ω*NopAA&NopD* inoculation was associated with a reduction in the numbers of infection threads and nodules, although these effects did not differ significantly from the phenotypes observed following *NopAA* mutant or *NopD* mutant inoculation. This suggests that NopAA and NopD serve as redundant regulators of nodulation or that crosstalk between the signaling pathways downstream of these effectors shapes nodule formation. Both NopAA and NopD ultimately impact nodulation via their effects on rhizobial infection, and while both of these T3Es can promote nodulation, neither appears to be required for nodulation.

T3Es can exert their functions when secreted into plants, whereupon host proteins can respond to or interact with these effectors. Both genetic and RNA-seq analyses have been utilized to identify the downstream response pathways and interacting proteins associated with these T3Es [21,24,57,58]. Here, RNA-seq analyses revealed that NopAA can induce substantial numbers of DEGs enriched in the MAPK pathway, in line with prior data related to rhizobia infection. NopAA does not induce necrosis in tobacco leaves [24], and it impacts *GmCDPK28* and *GmWRKY33* expression [42], suggesting that it may play a role in shaping plant defense responses through the regulation of PTI. NopAA was also found to influence many phenylpropanoid biosynthesis-related genes. Phenylpropanoid metabolic pathway-derived lignin is a primary component of cell walls in plants, and NopAA-mediated cellulose hydrolysis can cause cell wall damage and impact lignin metabolism [24]. The C-terminal region of NopD from *S. fredii* HH103 harbors a critical functional domain with a sequence similar to that of Bel2-5 and XopD [45]. Bel2-5 functions as a promoter of nodulation through its ability to regulate cytokinin biosynthesis and ethylene biosynthesis [28], whereas XopD plays a role in shaping host salicylic acid, gibberellic acid, abscisic acid, and ethylene signaling pathway activity [46]. NopD was also found to impact large numbers of genes enriched in the plant hormone signal transduction signaling pathway. In tobacco, NopD can trigger ETI-like programmed cell death [44], potentially consistent with its role in the plant–pathogen interaction signaling pathway. Much like the HH103Ω*NopAA* strain, inoculation with the HH103Ω*NopD* strain was also herein found to result in the differential expression of a large number of phenylpropanoid biosynthesis-related genes. Altered cell wall modification- and xyloglucan metabolism-related gene expression has also been observed in response to bel2-5 deletion mutants [28]. When utilizing HH103 inoculation as the comparator, many overlapping DEGs were identified between *NopAA*-mutant-inoculated and *NopD*-mutant-inoculated roots that were related to phenylpropanoid biosynthesis. While *NopAA* mutant inoculation did not result in the differential expression of many plant hormone signal transduction-related DEGs, a large number of hormone signal transduction-related DEGs were identified when assessing the overlapping DEGs between *NopAA*-mutant-inoculated and *NopD*-mutant-inoculated roots. These results are consistent with potential functional redundancy between NopD and NopAA with respect to their effects on hormone signal transduction. The greatest proportion of overlapping DEGs between *NopAA*-mutant-inoculated and *NopD*-mutant-inoculated roots were enriched in the plant–pathogen interaction pathway, and a large number of overlapping DEGs were noted with respect to MAPK pathway enrichment. Many of these overlapping DEGs were also enriched in XopD-associated pathways, suggesting that there is a degree of gene network redundancy or synergistic regulatory efficacy between NopAA and NopD with respect to the regulation of interactions between soybean plants and HH103 rhizobia. HH103Ω*NopAA&NopD* inoculation did not further modulate the expression of these overlapping DEGs, nor did it further inhibit nodulation. This may partially explain why HH103&*NopAA&NopD* inoculation did not further inhibit nodulation.

Inoculation with HH103Ω*NopAA&NopD* was found to specifically elicit the greatest number of DEGs as compared to HH103, suggesting that when *NopAA* and *NopD* are both mutated, a greater number of soybean host genes are engaged to respond as a means of maintaining symbiotic nodulation. Using a WGCNA approach to identify soybean genes that were specifically responsive to HH103Ω*NopAA&NopD* inoculation, *GmNAC27* was identified as ultimately confirmed to be significantly induced by this mutant strain in qRT-PCR analyses. GmNAC27 is a root-specific member of the plant-specific NAC transcription factor family that shares a high degree of homology with the *A. thaliana* AtNAC072 and AtNAC3 proteins. AtNAC072, also referred to as RESPONSIVE TO DESICCATION 26 (RD26), can enhance the ABA-dependent drought tolerance of plants. *AtNAC072* is reportedly upregulated in response to PGN, LPS, and flg22 treatment, suggesting that ANAC072 can respond to MAMP signaling [59]. AtNAC3 serves as a promoter of phytohormone synthesis that can bolster anti-pathogen defenses while repressing growth [60]. Infection with pathogens can reduce lncRNA *SABC1* accumulation, thus alleviating its ability to repress *AtNAC3* expression such that this gene is upregulated. When expressed, AtNAC3 can bind the promoter region upstream of *ICS1*, thereby promoting the upregulation of this key salicylic acid biosynthesis-related gene. *GmSIN* is a closely related gene cloned from the Shengdou 9 soybean cultivar that reportedly enhances soybean salt tolerance [61]. Here, *GmNAC27* overexpression in HH103-inoculated hairy roots was found to promote nodulation, suggesting that GmNAC27 serves as a positive regulator of nodule formation. The nodulation ability of *GmNAC27*-overexpressing hairy roots inoculated with HH103Ω*NopAA&D* did not differ significantly from that of control plants, while nodulation was significantly inhibited in *GmNAC27*-RNAi hairy roots inoculated with HH103Ω*NopAA&D*. This suggests that the absence of both NopAA and NopD, which positively regulate nodulation, may result in the upregulation of *GmNAC27* to facilitate the establishment of symbiosis. With respect to the mechanisms responsible for the upregulation of *GmNAC27*, it may be that host plants respond by overexpressing *GmNAC27* to maintain some level of nodulation, or it may be that NopAA and NopD synergistically repress *GmNAC27* such that it is upregulated when both of these effectors are absent. GmNAC27 was found to primarily impact the expression of the defense marker genes *GmPR1* and *GmPR5*, suggesting that it may shape defense response activity in the context of the establishment of symbiosis. However, further experiments will be essential to test these hypotheses. The construction of *GmNAC27* transgenic soybean plants will enable experiments aimed at directly confirming the functional role of GmNAC27 in the context of symbiosis, and efforts to screen for relevant genetic variations in different soybean populations may enable the breeding of soybean varieties with superior nitrogen-fixing efficiency.

## 4. Materials and Methods

### 4.1. Strains, Vectors and Primers

*Sinorhizobium fredii* HH103 (hereafter referred to as HH103) and mutants thereof were used to conduct the present study, as was *Escherichia coli* DH5α. HH103 and mutant strains were cultured with TY medium containing appropriate antibiotics at 28 °C, while *E. coli* were cultured in LB medium containing appropriate antibiotics at 37 °C. All antibiotics were used at a concentration of 50 μg/mL. Plasmids and primers used to conduct the present study are presented in Tables S1 and S2.

### 4.2. HH103ΩNopAA&NopD Mutant Strain Construction

Triparental hybridization was employed for HH103Ω*NopAA&D* mutant construction by first constructing the HH103Ω*NopAA* mutant [24], followed by the *NopD* mutation to yield HH103Ω*NopAA&D.*

### 4.3. Infection Event Analyses

SN14 plants were inoculated with HH103 and mutant strains thereof encoding the *GUS* reporter gene. At 36 h post-inoculation, roots were harvested, and GUS staining was performed by soaking in 1 mg ml^−1^ X-Gluc solution containing 100 mM potassium phosphate buffer (pH 7.0), 1 mM potassium ferricyanide, 1 mM potassium ferrocyanide, and 10 mM EDTA at 37 °C for 12 h. Then, 70% alcohol was used to decolorize stained roots, after which a total of 10 1 cm long lateral roots were collected from each plat for confocal imaging (Zeiss LSM700, Oberkochen, Germany). Three biological replicates, each consisting of 20 plants, were used for these analyses.

### 4.4. Nodulation Test

Cl_2_ was used to sterilize the surfaces of SN14 seeds for 12 h, after which they were sown in autoclaved vermiculite. Seedlings were then cultivated in a greenhouse (light/dark: 16 h/8 h, 25 °C) and routinely irrigated with F nutrient solution. HH103, *NopAA* mutant, *NopD* mutant, and *NopAA&D* mutant strains were cultured in liquid TY medium until reaching an OD_600_ of 0.6–0.8, at which time 10 mM MgSO_4_ solution was used to wash away the culture medium and to adjust the OD_600_ to 0.2.

Soybean roots were inoculated with rhizobia during the Vc phase, and nodule numbers and dry weight were assessed on day 28 post-inoculation. ANOVAs were used to test for significant differences among groups. Three biological replicates each consisting of 20 plants were analyzed for these experiments.

Nodule cross-sections were analyzed by embedding mature nodules (28 days post-inoculation) in paraffin. These nodules were then deparaffinized, stained using toluidine blue, and imaged under a light microscope (Olympus SZX16, Tokyo, Japan).

### 4.5. qRT-PCR

Following inoculation with appropriate rhizobia, soybean roots were harvested and TranZol Plant (Transgene Co., Beijing, China) was used to extract total RNA from these samples, followed by DNase I treatment to remove genomic DNA. cDNA was then prepared with the TransScript^®^ One-Step gDNA Removal and cDNA Synthesis SuperMix (Transgene Co.), after which the PerfectStart^TM^ Green One-Step qPCR SuperMix and appropriate primers were utilized for qRT-PCR analyses. The relative expression levels of symbiosis-related genes (*GmNIN*, *GmENOD40*, and *GmNSP1*), defense-related genes (*GmPR1*, *GmPR2*, and *GmPR5*) and candidate genes selected from RNA-seq were identified, and genes expression was normalized to the *GmUNK1* (*Glyma.12g020500*) reference gene [24].

### 4.6. RNA-Seq

The purity, integrity, and concentration of isolated RNA samples were assessed with NanoDrop, Agilent 2100 (Santa Clara, CA, USA)), and other appropriate instruments, after which cDNA library preparation was performed and Q-PCR was used for the quantification of library concentrations (effective concentration > 2 nM) to ensure library quality. Different libraries were then pooled based on the target downstream data volume followed by sequencing with an Illumina system (San Diego, CA, USA). All sequencing analyses were conducted by Biomarker (http://www.biomarker.com.cn/ (accessed on 15 March 2022)).

### 4.7. Subcellular Localization Analyses

Subcellular localization analyses were performed using 4-week-old *Nicotiana benthamiana* plants. Electroporation was used to transform *A. tumefaciens* EHA105 with the *CaMV35S-GmNAC27-GFP* plasmid, after which the *A. tumefaciens* culture was adjusted to an OD_600_ of 0.2 using infiltration buffer (10 mM MgCl_2_, 10 mM MES-KOH pH 5.6, 150 μM acetosyringone) and injected into the top leaves of tobacco plants. After 48 h, a Zeiss LSM 700 confocal laser scanning microscope (Zeiss, Oberkochen, Germany) was utilized to assess RFP and GFP fluorescence [21].

### 4.8. Soybean Hairy Root Transformation

Soybean hairy root transformation was performed using *A. rhizogenes* strain K599 containing *pSoy10-GmARP-GFP*, *pSoy10-GFP*, *pB7GWIWG2-GmARP-DsRed*, and *pB7GWIWG2-DsRed* [62]. Transgenic root selection was performed based on qRT-PCR results and the use of a portable fluorescent protein excitation light source (LUYOR), with positive hairy roots then being inoculated using HH103, *NopAA* mutant, *NopD* mutant, *NopAA&D* mutant, or MgSO_4_. Nodulation testing was performed at 28 days post-inoculation. Three independent experiments were utilized to assess nodulation phenotypes, with 20 biological replicates per experiment.

### 4.9. Phylogenetic Analyses

Those protein sequences exhibiting >75% similarity to Glyma.13G279900 were downloaded from Phytozome (https://phytozome.jgi.doe.gov/pz/portal.html (accessed on 20 April 2022)), imported into MEGA11, and used to conduct comparisons and construct phylogenetic trees which were processed with the Interactive Tree of Life (http://itol.embl.de/ (accessed on 20 April 2022)).

## 5. Conclusions

In summary, the present results revealed that NopAA and NopD have similar effects on *GmPR1*, *GmPR2*, and *GmPR5* expression in soybean. Nodule formation was influenced by the mutation of both *NopAA* and *NopD* as a consequence of changes in the incidence of rhizobial infection events. Nodulation tests did not reveal any significant differences in SN14 nodulation when comparing the HH103Ω*NopAA&D*, HH103Ω*NopAA*, and HH103Ω*NopD* strains. RNA-seq analyses further suggested the existence of potential signaling relationships between NopAA and NopD, while WGCNA and transgenic analyses revealed that soybean plants respond to the absence of both of these effectors by upregulating *GmNAC27*. Together, these results provide a foundation for research focused on T3Es signaling networks and offer support for efforts to breed high-yield soybean varieties with superior nitrogen fixation efficiency.

## Data Availability

The datasets presented in this study can be found in Supplementary Materials.

## Supplementary Materials

The following supporting information can be downloaded at: https://www.mdpi.com/article/10.3390/ijms242417498/s1.
