# Supplementary material for: NopAA and NopD Signaling Association-Related Gene GmNAC27 Promotes Nodulation in Soybean (Glycine max)
Source: Int J Mol Sci. 2023 Dec 15;24(24):17498. doi: 10.3390/ijms242417498 (PMC10744329; doi:10.3390/ijms242417498)
Supplement: Supplementary file 1 [file ijms-24-17498-s001.zip › Supplementary Figures.pdf]

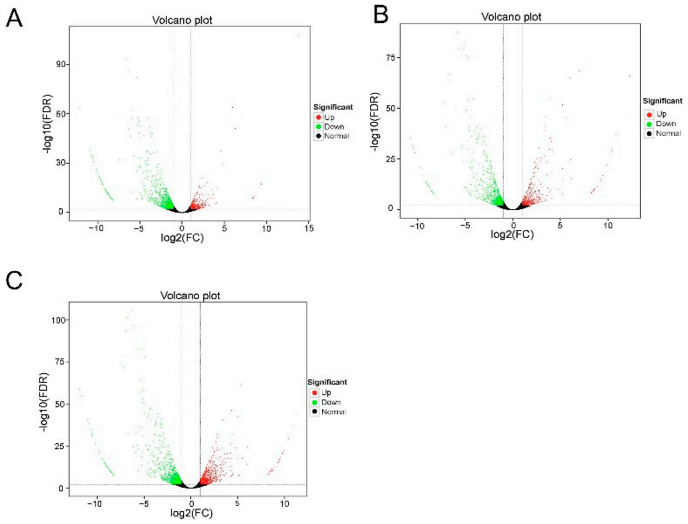

Figure S1. DEGs identified in SN14 roots following inoculation with different HH103 mutant strains relative to parental HH103 inoculation. (A-C) Volcano plots corresponding to DEGs respectively up-/down-regulated by HH103Ω*NopAA*, HH103Ω*NopD*, and HH103Ω*NopAA&D* relative to HH103.

A

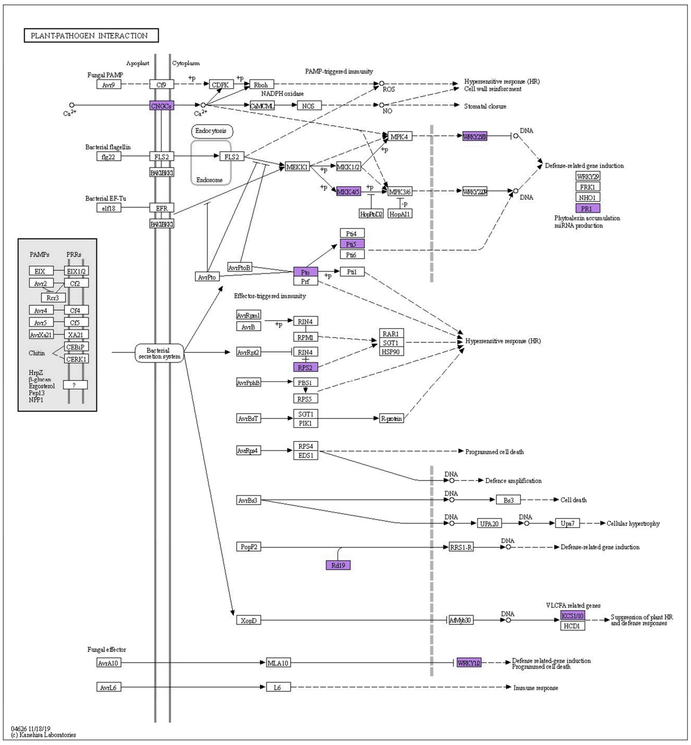

B

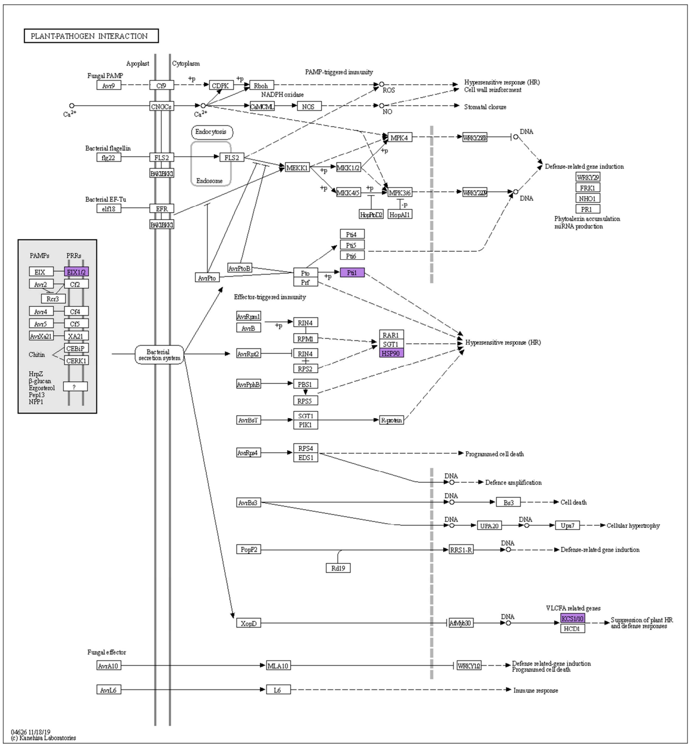

Figure S2. A KEGG pathway map for common DEGs shared between the NopAA vs. HH103 and NopD vs. HH103 comparisons enriched in plant-pathogen interaction. (A) A KEGG pathway map for common downregulated DEGs. (B) A KEGG pathway map for common upregulated DEGs.

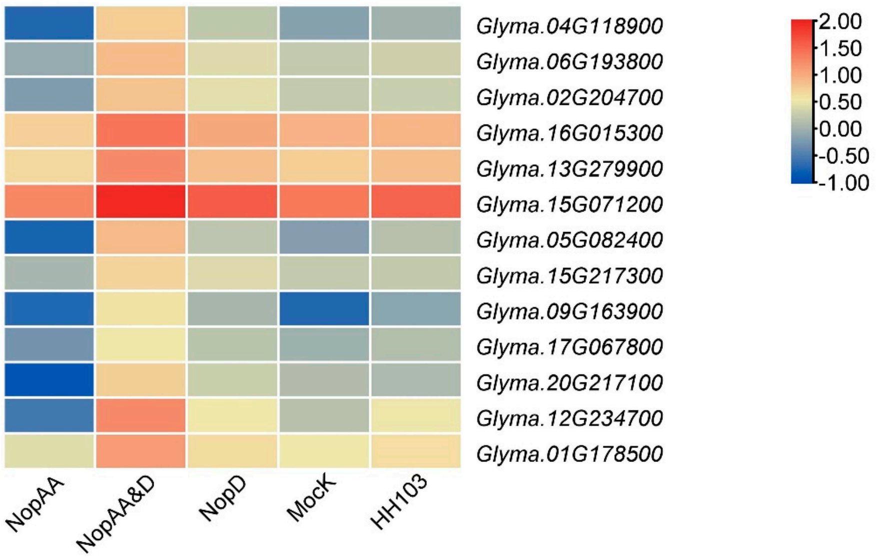

Figure S3. Hub genes heatmap.

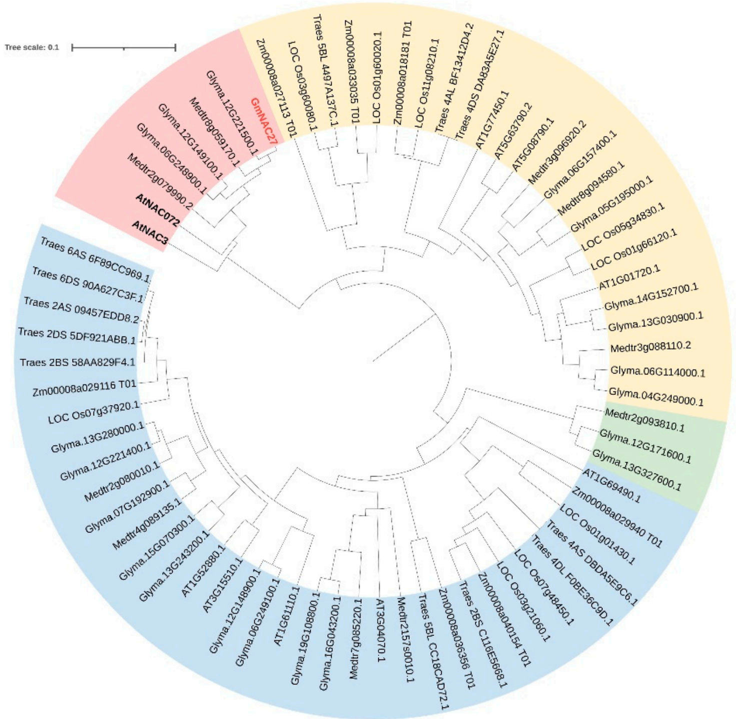

Figure S4. Phylogenetic tree analysis of GmNAC27(marked in red).

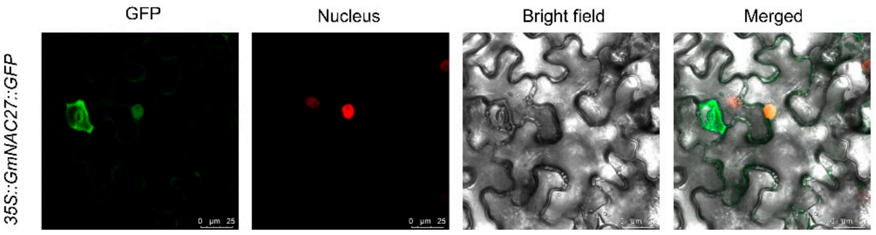

Figure S5. Subcellular localization analyses of GmNAC27 in tobacco. Scale bars:25 μm.

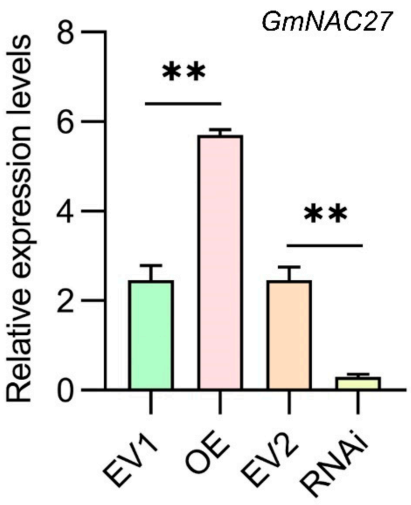

Figure S6. Relative GmNAC27 expression in transgenic hairy roots. EV1, Empty vector for *GmNAC27* overexpression; OE, *GmNAC27* overexpression vector under the control of CaMV35S; EV2, Empty vector for RNAi; RNAi, *GmNAC27* silencing. Significance was determined by Student's *t*-test (** $p<0.01$).

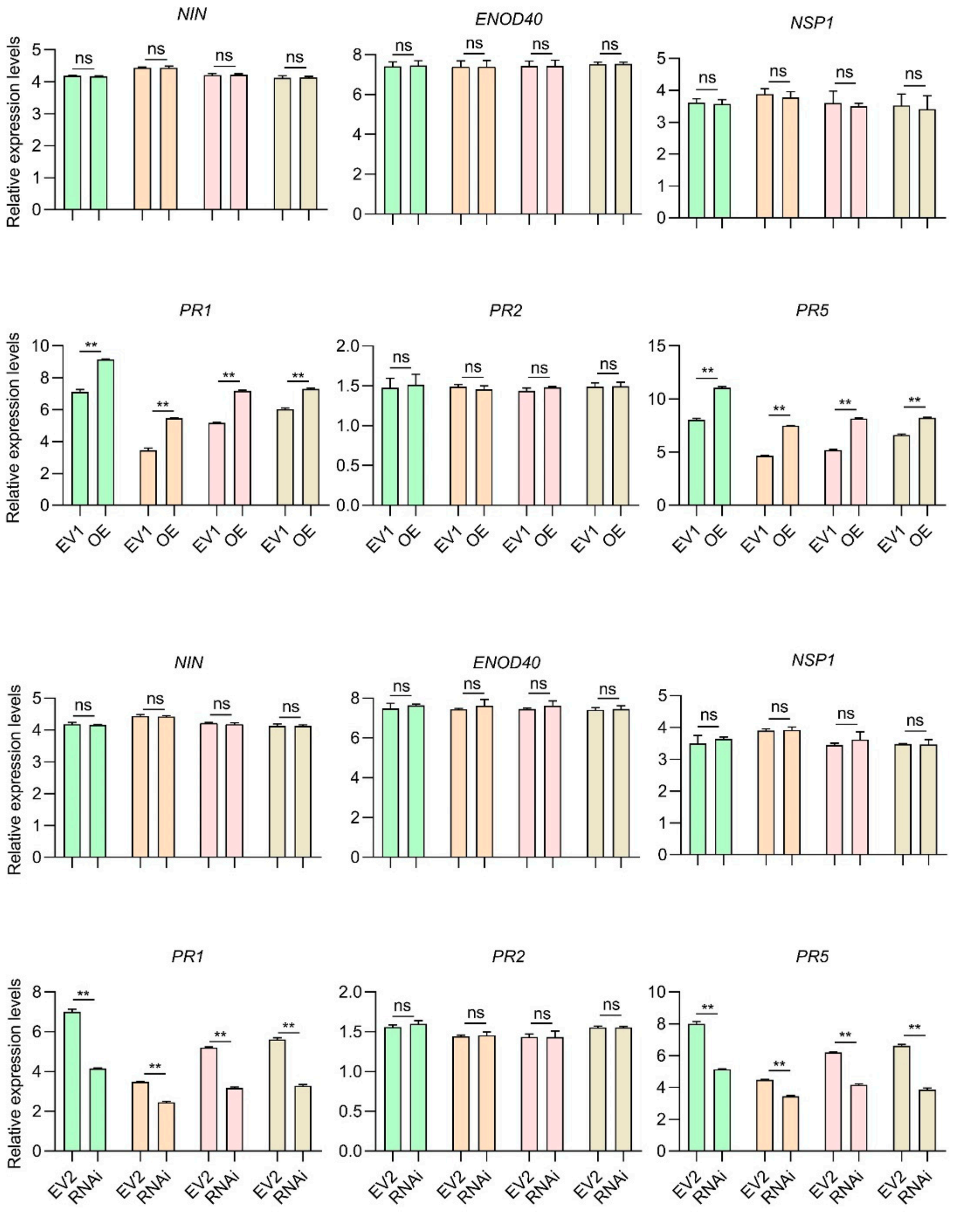

Figure S7. Relative maker gene expression levels in transgenic hairy roots. EV1, Empty vector for *GmNAC27* overexpression; OE, *GmNAC27* overexpression vector under the control of CaMV35S; EV2, Empty vector for RNAi; RNAi, *GmNAC27* silencing. Significance was determined by Student's *t*-test (** $p<0.01$, ns $p>0.05$).
