# Supplementary material for: NopAA and NopD Signaling Association-Related Gene GmNAC27 Promotes Nodulation in Soybean (Glycine max)
Source: Int J Mol Sci. 2023 Dec 15;24(24):17498. doi: 10.3390/ijms242417498 (PMC10744329; doi:10.3390/ijms242417498)
Supplement: Supplementary file 1 [file ijms-24-17498-s001.zip › Supplementary Tables.pdf]

Table S1. Primers used in this study

No.	Name of primer	Sequence 5'-3'	Annotation and Functions
1	*qNIN*	Fwd-GAAGGGTTTTGACGAGTTGTAC	qRT-PCR
		Rev-TGTGAGAGCTAAAGGCAAGTTA	qRT-PCR
2	*qENOD40*	Fwd-TGGCTATGTAGTGCTCATGTAG<br>Rev-GCAAAAGCTGGTAACTTCTCAA	qRT-PCR
3	*qNSP1*	Fwd-CACTGAATGCTCAAAGTGTTGA<br>Rev-GTTCCTCAACACTGTCAGAAAC	qRT-PCR
4	*qPR1*	Fwd-GGCCAATACGGGGAGAATCT<br>Rev-TCCAAACAACCTGAGTGTAATGC	qRT-PCR
5	*qPR2*	Fwd-TGAGAGTGGATGGCCTTCTT<br>Rev-TGTTTCACATTCCGAACCAA	qRT-PCR
6	*qPR5*	Fwd-AACGTGCCCATGGACTTTAG<br>Rev-CGGTTTTGAAGACAGTGCAA	qRT-PCR
7	*qGlyma.13G279900*	Fwd-GAGCTTCTCGTTCAGTATCTGT<br>Rev-CTTGTACAAGTCAATTTCGGCA	qRT-PCR
8	*qGlyma.04G118900*	Fwd-CTCTCAAGCTATGCAATGTGTC<br>Rev-TCACATTCCCAAAGCAATCTTG	qRT-PCR
9	*qGlyma.06G193800*	Fwd-CTGCTACAACAACTGGAAGAAC<br>Rev-TTGACTAATCAGGAGAACACCC	qRT-PCR
10	*qGlyma.02G204700*	Fwd-CCCGAAATATTGACATGTAACACG<br>Rev-TTGAAAAATTGACACCCTAGCC	qRT-PCR
11	*qGlyma.15G071200*	Fwd-TGCCAAAGAAATCTCAACGAAG<br>Rev-CTTTCAACGAGAGCAGAAACAA	qRT-PCR
12	*qGlyma.16G015300*	Fwd-GTCGTGGCTTTACTTTCCTTTT<br>Rev-TCAACTGAATGCAACTGTTCAC	qRT-PCR

13	*qGlyma.05G082400*	Fwd-GCTTCCATGAATCTGGCTAATG<br>Rev-TATTTGACAGTACGCGGATCAT	qRT-PCR
14	*qGlyma.15G217300*	Fwd-ATACCACAGTGTGTTTTGGAGT<br>Rev-CTATCAAGCAGCACAAGACAAG	qRT-PCR
15	*qGlyma.09G163900*	Fwd-AGCACTTCCTTTCATTTGGTTC<br>Rev-GCTGCACTAATTTCGTTAACCA	qRT-PCR
16	*qGlyma.17G067800*	Fwd-CTTCTGGAAGTGGATGAGAGTT<br>Rev-TTCTCATTTATCTCCCTCGCAA	qRT-PCR
17	*qGlyma.20G217100*	Fwd-TTTCAAATACCCTAATTCGCGC<br>Rev-GTACTTGGCGAAATGGAGAAAG	qRT-PCR
18	*qGlyma.12G234700*	Fwd-GAGCTACCGTATGTCGATGTAT<br>Rev-TCTAATCTCTGAGTGGGATCCT	qRT-PCR
19	*qGlyma.01G178500*	Fwd-AAAGAGTTGCATAACGTTCTCG<br>Rev-ATTCTCTTCTTACCGACTCGTG	qRT-PCR
20	*qUKN1*	Fwd-TGGTGCTG CCGCTATTTACTG<br>Rev-GGTGGAAGGAACTGCTAACAAT	Reference gene for qRT-PCR
21	*NAC27*	Fwd-ATGGGAGTTCCAGAGAAAGA<br>Rev-ATTTCTGAACCCGAACCCG	Clone *GmNAC27* fragment
22	*RNAi-NAC27*	Fwd-TCTTCCAAGTAAAGCGATTT<br>Rev- AAATCGCTTTACTTGGAAGA	Clone *GmNAC27-RNAi* fragment into pGWC for gene silencing

Table S2. Information of Strains and Vectors

Strain	Relevant characteristics	Reference
Escherichia coli		
DH5α	supE44 lacY169 (80lacZM15) hsdR17 recA1 endA1 gyrA96 thi-1 relA1	Transgene (Transgene Biotech Co., Beijing, China)
Agrobacterium rhizogenes		
EHA105	C58 (rifR) Ti pEHA105 (pTiBo542DT-DNA) (strepR) Succinamopine	Transgene (Transgene Biotech Co., Beijing, China)
K599	pRi2659 agrobacterium-type Ri plasmid,Strep^r	Beyotime (Beyotime Biotech Co., Shanghai, China)
***Rhizobium* strains**		
HH103	Broad host range bacterium isolated from nodules of Glycine max , Rif^r	Wang J, Wang J et al[1]
HH103ΩNopAA	HH103 insertion mutated containing an Kanamycin resistance gene insertion at position downstream 17bp of start codon of NopAA nucleotide sequence, Rif^r, Kan^r	Wang J, Ma C et al[1]
HH103ΩNopD	HH103 insertion mutated containing an Kanamycin resistance gene insertion at position downstream 9bp of start codon of *NopD* nucleotide sequence, Rif^r, Kan^r	Wang J, Wang J et al[1]
HH103ΩNopAA&D	HH103ΩNopAA insertion mutated containing an spectinomycin resistance gene insertion at *NopD* nucleotide sequence, Rif^r,Kan^r ,Spec^r	This work
HH103ΩTtsI	HH103 insertion mutated containing a Kanamycin resistance gene insertion at position downstream 8bp of start codon of *TtsI* nucleotide sequence, Rif^r, Kan^r	Wang J, Wang J et al[1]
**Plasmids**		
pGWC	Entry clone vector, Cm^r	Wang J, Wang J et al[1]

Note: Rifampicin (Rif^r) Kanamycin (Kan^r) Chloramphenicol (Cm^r) Spectinomycin(Spec^r) Streptomycin(Strep^r)

References cited in Table S2

[1] Wang J, Wang J, Ma C, Zhou Z, Yang D, Zheng J, Wang Q, Li H, Zhou H, Sun Z, Liu H, Li J, Chen L, Kang Q, Qi Z, Jiang H, Zhu R, Wu X, Liu C, Chen Q, Xin D. QTL Mapping and Data Mining to Identify Genes Associated With the Sinorhizobium fredii HH103 T3SS Effector NopD in Soybean. Front Plant Sci. 2020 May 19;11:453.

Table S3. The candidate genes annotation

No.	Gene ID	Annotations on phytozome
1	*GLYMA.04G118900*	NONE
2	*GLYMA.06G193800*	GIBBERELLIN-REGULATED PROTEIN 12-RELATED
3	*GLYMA.02G204700*	SERINE CARBOXYPEPTIDASE 24-RELATED
4	*GLYMA.16G015300*	RNA RECOGNITION MOTIF RNP-1 DOMAIN-CONTAINING PROTEIN
5	*GLYMA.13G279900*	NAC DOMAIN-CONTAINING PROTEIN 19-RELATED
6	*GLYMA.15G071200*	CATECHOL OXIDASE / TYROSINASE
7	*GLYMA.05G082400*	LEUCINE-RICH REPEAT-CONTAINING PROTEIN
8	*GLYMA.15G217300*	DNA-3-METHYLADENINE GLYCOSYLASE I / DNA-3-METHYLADENINE GLYCOSIDASE I
9	*GLYMA.09G163900*	TRYPSIN AND PROTEASE INHIBITOR
10	*GLYMA.17G067800*	LPHA, ALPHA-TREHALOSE-PHOSPHATE SYNTHASE 11-RELATED
11	*GLYMA.20G217100*	PLASTOCYANIN-LIKE DOMAIN
12	*GLYMA.12G234700*	TRYPSIN AND PROTEASE INHIBITOR
13	*GLYMA.01G178500*	UNCHARACTERIZED CONSERVED PROTEIN
